# Sarcopenia is associated with poor prognosis after chemoradiotherapy in patients with stage III non-small-cell lung cancer: a retrospective analysis

**DOI:** 10.1038/s41598-021-91449-z

**Published:** 2021-06-04

**Authors:** Kuniaki Katsui, Takeshi Ogata, Soichi Sugiyama, Kotaro Yoshio, Masahiro Kuroda, Takao Hiraki, Katsuyuki Kiura, Yoshinobu Maeda, Shinichi Toyooka, Susumu Kanazawa

**Affiliations:** 1 Department of Proton Beam Therapy, Okayama University Graduate School of Medicine, Dentistry and Pharmaceutical Sciences, 2-5-1 Shikata-cho, Kita-ku, Okayama, 700-8558 Japan; 2 Department of Radiology, Iwakuni Clinical Center, 1-1-1 Atagomachi, Iwakuni, Yamaguchi 740-8510 Japan; 3 Department of Radiology, Okayama University Hospital, 2-5-1 Shikata-cho, Kita-ku, Okayama, 700-8558 Japan; 4 Department of Radiological Technology, Graduate School of Health Sciences, Okayama University, 2-5-1 Shikata-cho, Kita-ku, Okayama, 700-8558 Japan; 5 Department of Radiology, Okayama University Graduate School of Medicine, Dentistry and Pharmaceutical Sciences, 2-5-1 Shikata-cho, Kita-ku, Okayama, 700-8558 Japan; 6 Department of Allergy and Respiratory Medicine, Okayama University Hospital, 2-5-1 Shikata-cho, Kita-ku, Okayama, 700-8558 Japan; 7 Department of Hematology, Oncology and Respiratory Medicine, Okayama University Graduate School of Medicine, Dentistry, and Pharmaceutical Sciences, 2-5-1 Shikata-cho, Kita-ku, Okayama, 700-8558 Japan; 8 Department of General Thoracic Surgery and Breast and Endocrinological Surgery, Okayama University Graduate School of Medicine, Dentistry and Pharmaceutical Sciences, 2-5-1 Shikata-cho, Kita-ku, Okayama, 700-8558 Japan

**Keywords:** Medical research, Oncology

## Abstract

We intended to investigate whether muscle and adipose masses were associated with prognosis among patients with stage III non-small-cell lung cancer (NSCLC) who were undergoing chemoradiotherapy (CCRT). We retrospectively explored data of patients with stage III NSCLC who underwent definitive CCRT (≥ 60 Gy) between January 2004 and March 2018 at our hospital. We examined the relationship of overall survival (OS) with body mass index (BMI), skeletal muscle index (SMI), psoas muscle index (PMI), visceral adipose tissue index (VAI), subcutaneous adipose tissue index (SAI), and visceral-to-subcutaneous adipose tissue area ratio (VSR) using log-rank tests for the univariate analysis and Cox proportional hazard models for the multivariate analysis. Overall, 16, 32, and 12 patients had stage IIIA, IIIB, and IIIC NSCLC, respectively. The total radiotherapy dose ranged from 60 Gy/30 fractions to 66 Gy/33 fractions. In the univariate analysis, the performance status (PS), BMI, and SMI were associated with OS, whereas the PMI, VAI, SAI, and VSR were not. In the multivariate analysis, the PS and SMI were associated with OS. The hazard ratios and 95% confidence intervals were 2.91 and 1.28–6.64 for PS, and 2.36 and 1.15–4.85 for SMI, respectively. The 1, 3, and 5-year OS rates were 92.1%, 59.6%, and 51.0% in patients with high SMI, and 63.6%, 53.8%, and 17.9% in patients with low SMI, respectively. The SMI correlated with prognosis in our study population, whereas adipose mass did not. Therefore, sarcopenia should be considered while predicting the OS in such patients.

## Introduction

The incidence of lung cancer is increasing worldwide, with 1.6 million cases reported in 2008 and 2.1 million in 2018^1,2^. The age-standardized 5-year overall survival (OS) rate associated with lung cancer is as low as 10–20%, and the disease remains associated with poor prognosis^3^; therefore, there is an urgent need to develop effective prognostic and management strategies.

Sarcopenia, first defined by Rosenberg^4^, is the loss of muscle mass in association with aging, decreased activity levels, malnutrition, organ failure, and diseases such as malignancies^5,6^. An association between sarcopenia and adverse health effects, including falls, disability, hospitalization, long-term care placement, poor quality of life, and mortality has been confirmed^6^. Sarcopenia is also associated with poor prognosis after treatment for malignancies, including lung cancer^7–13^. Further, an association between sarcopenia and poor prognosis in surgical cases, especially those involving early-stage lung cancer, has been demonstrated in many studies^12,13^. In a study by Baracos et al., 46.8% of the patients with stage III–IV non-small-cell lung cancer (NSCLC) were diagnosed with sarcopenia^14^. Concurrent chemoradiotherapy (CCRT) is among the standard treatments for stage III lung cancer^15,16^. Few studies have focused on the association between prognosis and sarcopenia in patients with NSCLC who were treated with chemoradiotherapy. Bowden et al. demonstrated that muscle attenuation, and not muscle mass, was associated with long-term survival in patients with lung cancer, including those with small-cell lung cancer and NSCLC, who underwent chemoradiotherapy^17^. Kiss et al. did not identify an association between muscle mass or muscle attenuation and OS in patients with NSCLC who underwent CCRT^18^. Existing evidence on the association between sarcopenia and OS in patients treated with CCRT for stage III NSCLC is insufficient.

Body mass index (BMI)-determined obesity is a poor prognostic factor after treatment in most cancer types^19,20^. However, the relationship between obesity and OS after treatment in patients with lung cancer is controversial^19,20^. In a meta-analysis, Gupta et al. showed that patients with obesity and overweight had lower lung cancer mortality rates than those with normal BMI^21^. Dahlberg et al. showed that patients with advanced NSCLC and obesity who received chemotherapy had better OS than those without obesity in the early stages of their study^22^. In locally advanced NSCLC settings, Lam et al. showed that the OS of patients who were obese was better than that of those who were normal-weight^23^. In the aforementioned studies, obesity was assessed based on BMI alone without using computed tomography (CT). In recent years, sarcopenia and adiposity have been measured based on muscle and fat masses using CT, which is necessary for diagnosing and staging cancer^7,11,17,18,24–26^. Although BMI is not a poor prognostic factor, the visceral-to-subcutaneous adipose tissue mass, in addition to muscle mass, is an independent poor prognostic factor in patients with hepatocellular carcinoma and intrahepatic cholangiocarcinoma^25,26^. Because weight includes components besides muscles, the measurement of fat mass in addition to muscle mass using CT is desirable. However, no studies to date have examined the association between fat mass and OS in patients with NSCLC who were treated with CCRT.

The identification of the level of risk before treatment and provision of appropriate interventions for sarcopenia and obesity may improve patient prognosis. Therefore, we intended to investigate whether CT-determined muscle and fat masses were prognostic factors in patients with stage III NSCLC who have undergone CCRT.

## Patients and methods

### Patients and treatment

We retrospectively explored data of patients with stage III NSCLC who underwent definitive CCRT (≥ 60 Gy) between January 2004 and March 2018 at our hospital. This study included patients for whom pre-CCRT digital plain CT of the third lumbar vertebra (L3) was available in the picture archiving and communication systems. Patients who underwent treatment for NSCLC before CCRT and those who received preoperative CCRT with chest surgery before recurrence were excluded. Staging was determined using the tumor-node-metastasis classification, 8th edition. We examined the patients’ histology, smoking history, performance status (PS), location, laterality, and forced expiratory volume in 1 s. The indication of CCRT was determined by consensus of board-certified respiratory physicians and radiation oncologists. The chemotherapy regimen was determined by board-certified respiratory physicians, and details regarding radiotherapy were finally determined by board-certified radiation oncologists. Chemotherapy mainly involved the administration of cisplatin plus docetaxel; tegafur/gimeracil/oteracil was used for vulnerable patients. All patients were treated with three-dimensional conformal radiotherapy. Details regarding the three-dimensional radiotherapy which was employed have been described elsewhere^27^. The gross tumor included the primary tumor and the clinically diagnosed metastatic lymph node. The margin for the clinical target volume was 5–10 mm, and subcarinal and ipsilateral hilar nodal stations were included in cases of elective nodal irradiation. The internal margin was determined using fluoroscopic images, and the planning target volume margin was 5–10 mm. We used a 10 MV photon beam generated from a linear accelerator (Primus or ONCOR, or Mevatron, Canon Medical Systems, Tochigi, Japan).

All procedures were performed according to the ethical standards set out in the 1964 Declaration of Helsinki and subsequent amendments. This study was approved by our institutional review board of Okayama University Graduate School of Medicine, Dentistry and Pharmaceutical Sciences and Okayama University Hospital (approval number 1809-018). Written informed consent for CCRT was obtained from all patients before treatment. The choice to opt out was provided through notifications displayed on the hospital’s website and in the outpatient ward before starting the study.

### Image analysis

**Figure 1:**
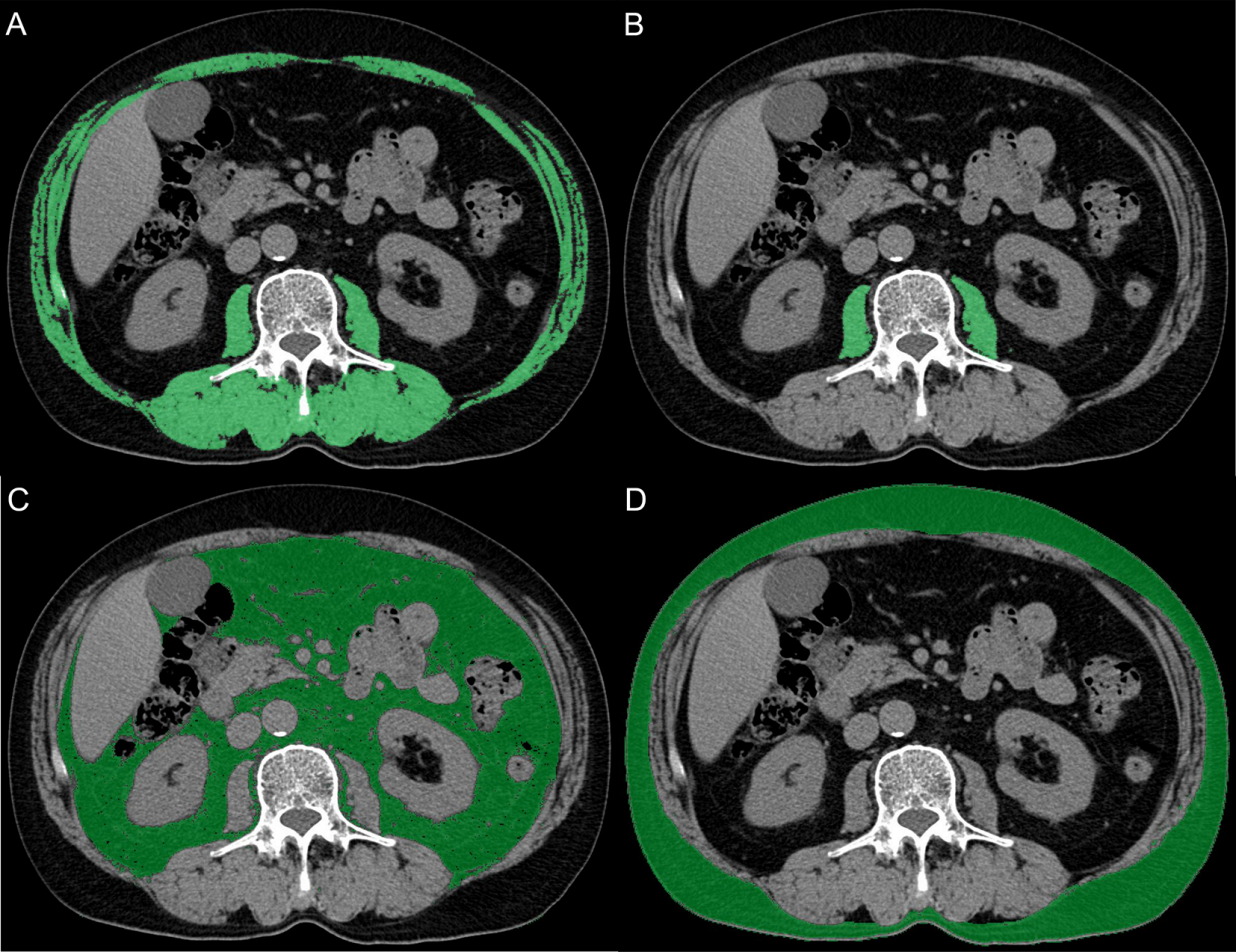
Computed tomography images of the third lumbar vertebra for each area. **(a)** Green highlights indicate the skeletal muscle area for the skeletal muscle index. **(b)** Green highlights indicate the psoas muscle area for the psoas muscle index. **(c)** Green highlights indicate the visceral adipose tissue area for the visceral adipose index. **(d)** Green highlights indicate the subcutaneous adipose tissue area for the subcutaneous adipose index.

Abdominal CT data, which were obtained from the institution’s picture archiving and communication systems, were transferred to a workstation computer (SYNAPSE VINCENT version 5.5, FUJIFILM Medical Co., Ltd., Tokyo, Japan). Plain CT was used to examine the vertebra at the L3 level, and contours were extracted automatically by setting the Hounsfield unit (HU) thresholds within a certain range. According to previous studies, the HU thresholds were set from − 29 to + 150 for the skeletal and psoas muscles, − 150 to − 50 for visceral adipose tissues, and − 190 to − 30 for subcutaneous adipose tissues^7,24^. Skeletal muscles included all muscles at the L3 level. Areas that were not obviously targeted muscle or adipose tissues were deleted by manual manipulation. Figure 1A–D illustrate an example for each of the muscle and adipose tissues. These four areas (cm^2^) were divided by the square of height (m) to obtain the skeletal muscle index (SMI), psoas muscle index (PMI), visceral adipose index (VAI), and subcutaneous adipose index (SAI)^25,28^. Muscle attenuation (MA), as a measure of muscle quality, was defined as the mean of the HU value of the SMI; furthermore, the visceral-to-subcutaneous adipose ratio (VSR) was calculated^7,25^.

### Evaluation and statistical analyses

Cutoff values for continuous variables were calculated based on individual time-dependent receiver operating characteristics^29^. According to previous reports^26^, we calculated the cutoff values for men and women and divided them into two groups. The survival curves were obtained using the Kaplan–Meier method. We examined the relationship between the aforementioned parameters and OS using log-rank tests for the univariate analysis and Cox proportional hazard models for the multivariate analysis. Statistical significance was determined at p-values < 0.05 (two-sided). R software version 3.5.1 and additional package of survival ROC version 1.0.3 (R Foundation for Statistical Computing, Vienna, Austria) were used for all statistical analyses.

## Results

This study included 60 eligible patients. Table 1 shows the patients’ characteristics. Overall, 16 patients had stage IIIA NSCLC, 32 had stage IIIB, and 12 had stage IIIC. The follow-up period was 29.1 months (2.4–121.4 months) from the initiation of CCRT. The total radiotherapy doses were 60 Gy/30 fractions in 59 patients and 66 Gy/33 fractions in one patient. Details regarding the chemotherapy regimen are shown in Table 1. The median BMI, SMI, PMI, MA, VAI, SAI, and VSR were 21.7 kg/m^2^ (range: 14.2–34.1 kg/m^2^), 43.1 cm^2^/m^2^ (range: 23.5–64.8 cm^2^/m^2^), 5.1 cm^2^/m^2^ (range: 2.7–8.6 cm^2^/m^2^), 35.9 HU (range: 8.6–49.0 HU), 27.0 cm^2^/m^2^ (0.7–134.4 cm^2^/m^2^), 28.7 cm^2^/m^2^ (0.0–127.1 cm^2^/m^2^), and 1.0 (range: 0.1–25.8), respectively.

**Table 1:** Patient characteristics.

			%
Sex	Male	51	85
	Female	9	15
Age (years)	Median (range)	66 (35–87)	–
T stage	1	6	10
	2	12	20
	3	5	8
	4	33	55
	X	4	7
N stage	0	3	5
	1	5	8
	2	26	43
	3	26	43
Clinical stage	IIIA	16	27
	IIIB	32	53
	IIIC	12	20
Histology	Adenocarcinoma	26	43
	Squamous cell carcinoma	28	47
	Non-small-cell carcinoma	6	10
Smoking history^a^	Never	5	8
	Former	31	52
	Current	22	37
ECOG-PS^a^	0	21	35
	1	36	60
Lobe^a^	Upper	45	75
	Middle	1	2
	Lower	11	18
Laterality	Right	31	52
	Left	26	43
FEV1 (l)^a^	Median (range)	2.15 (0.86–4.11)	–
Chemotherapy	Standard	49	82
	Cisplatin + Docetaxel	40	67
	Cisplatin + Vinorelbine + Nimotuzumab	1	2
	Cisplatin + Etoposide	1	2
	Cisplatin + Tegafur/Gimeracil/Oteracil	5	8
	Carboplatin + Paclitaxel	2	3
	Reduced	11	18
	Cisplatin	1	2
	Tegafur/Gimeracil/Oteracil	10	17
Radiation dose (Gy)	Median (range)	60 (60–66)	–
BMI^a^ (kg/m^2^)	Median (range)	21.7 (14.2–34.1)	–
SMI (cm^2^/m^2^)	Median (range)	43.1 (23.5–64.8)	
PMI (cm^2^/m^2^)	Median (range)	5.1 (2.7–8.6)	–
MA (HU)	Median (range)	35.9 (8.6–49.0)	–
VAI (cm^2^/m^2^)	Median (range)	27.0 (0.7–134.4)	–
SAI (cm^2^/m^2^)	Median (range)	28.7 (0.0–127.1)	–
VSR	Median (range)	1.0 (0.1–25.8)	–

*ECOG-PS* Eastern Cooperative Oncology Group performance status, *FEV1* forced expiratory volume in 1 s, *BMI* body mass index, *MA* mean muscle attenuation, *SMI* skeletal mass muscle index, *PMI* psoas muscle index, *VAI* visceral adiposity index, *SAI* subcutaneous adiposity index, *VSR* visceral-to-subcutaneous fat ratio.

^a^These factors have missing values.

Table 2 shows the relationship between clinicopathologic factors and sarcopenia (low SMI). Sarcopenia was significantly associated with lower BMI (p = 0.017). Table 3 shows the cutoff values and the results of the univariate and multivariate analyses of factors associated with OS. The female/male cutoff BMI, SMI, PMI, MA, VAI, SAI, and VSR values determined from the time-dependent receiver operating characteristic curve were 17/25 kg/m^2^, 24/43 cm^2^/m^2^, 4.7/7.3 cm^2^/m^2^, 9/36 HU, 97/40 cm^2^/m^2^, 127/28 cm^2^/m^2^, and 0.8/0.6, respectively. The PS, SMI, and BMI were significant prognostic factors associated with OS in the univariate analysis (p = 0.004, 0.01, and 0.03), whereas the PMI, MA, VAI, SAI, and VSR were not (p = 0.4, > 0.99, 0.1, 0.09, and 0.1, respectively). In the multivariate analysis, the PS and SMI were significant prognostic factors associated with OS (p = 0.011 and 0.020, respectively). The hazard ratios and 95% confidence intervals (CIs) were 2.91 and 1.28–6.64 for the PS, 2.36 and 1.15–4.85 for the SMI, respectively. Since BMI and SMI are significantly correlated, we excluded BMI from the multivariate analysis according to previous reports^30,31^.

**Table 2:** Relationship between clinicopathologic factors and sarcopenia.

Factor		With sarcopenia (low SMI)	%	Without sarcopenia (high SMI)	%	p value
Sex	Female	1	5	8	21	0.14
	Male	21	95	30	79	
Age (years)	< 66	8	36	20	53	0.29
	≥ 66	14	64	18	47	
T stage	X, 1–2	5	23	17	45	0.1
	3–4	17	77	21	55	
N stage	0–1	6	27	2	5	0.042
	2–3	16	73	36	95	
Clinical stage	IIIA	6	27	10	26	0.52
	IIIB	10	45	22	58	
	IIIC	6	27	6	16	
Histology	Adenocarcinoma	8	36	18	47	0.43
	Others	14	64	20	53	
Smoking history^a^	Never/former	15	68	21	53	0.58
	Current	7	32	15	47	
ECOG-PS^a^	0	7	32	14	40	0.58
	1	15	68	21	60	
Laterality	Right	6	73	25	29	0.002
	Left	16	27	10	71	
Lobe	Upper/middle	18	82	31	82	> 0.99
	Lower	4	18	7	18	
FEV1 (l)^a^	< 2.2	8	42	21	66	0.15
	≥ 2.2	11	58	11	34	
Chemotherapy	Standard	17	77	32	84	0.51
	Reduced	5	23	6	16	
BMI^a^	F: < 17, M: < 25	19	90	22	58	0.017
	F: ≥ 17, M: ≥ 25	2	10	16	42	
PMI	F: < 4.7, M: < 7.3	21	95	31	82	0.24
	F: ≥ 4.7, M: ≥ 7.3	1	5	7	18	
MA	F: < 9, M: < 36	11	50	13	34	0.28
	F: ≥ 9, M: ≥ 36	11	50	25	66	
VAI	F: < 97, M: < 40	18	82	24	63	0.15
	F: ≥ 97, M: ≥ 40	4	18	14	37	
SAI	F: < 127, M: < 28	15	68	19	50	0.19
	F: ≥ 127, M: ≥ 28	7	32	19	50	
VSR	F: < 0.8, M: < 0.6	8	36	11	29	0.58
	F: ≥ 0.8, M: ≥ 0.6	14	64	27	71	

*ECOG-PS* Eastern Cooperative Oncology Group performance status, *FEV1* forced expiratory volume in 1 s, *BMI* body mass index, *MA* mean muscle attenuation, *SMI* skeletal mass muscle index, *PMI* psoas muscle index, *VAI* visceral adiposity index, *SAI* subcutaneous adiposity index, *VSR* visceral-to-subcutaneous fat ratio, *CI* confidence interval.

^a^These variables have missing values.

**Table 3:** Univariate and multivariate analyses of factors associated with overall survival.

Factor		Number of deaths	Univariate analysis	Multivariate analysis	Hazard ratio
			*p*-value	*p*-value	(95% CI)
Sex	F	4/9	0.3	NE	–
	M	32/51			
Age (years)	< 66	16/28	0.6	NE	–
	≥ 66	20/32			
T Stage	X, 1–2	10/22	0.2	NE	–
	3–4	26/38			
N Stage	0–1	3/8	0.4	NE	–
	2–3	33/52			
Clinical stage	IIIA	6/16	0.3	NE	–
	IIIB	22/32			
	IIIC	8/12			
Histology	Adenocarcinoma	16/26	0.5	NE	–
	Others	20/34			
Smoking history^a^	Never/former	21/36	0.9	NE	–
	Current	13/22			
ECOG-PS^a^	0	8/21	0.004	0.011	2.91
	1	25/36			(1.28–6.64)
Laterality	Right	17/31	0.5	NE	–
	Left	18/26			
Lobe	Upper/middle	28/49	0.2	NE	–
	Lower	8/11			
FEV1 (l)^a^	< 2.2	17/29	0.2	NE	–
	≥ 2.2	13/22			
Chemotherapy	Standard	29/49	0.5	NE	–
	Reduced	7/11			
BMI^a^	F: < 17, M: < 25	28/41	0.03	NE	–
	F: ≥ 17, M: ≥ 25	7/18			
SMI	F: < 24, M: < 43	17/22	0.01	0.02	2.36
	F: ≥ 24, M: ≥ 43	19/38			(1.15–4.85)
PMI	F: < 4.7, M: < 7.3	31/52	0.4	NE	–
	F: ≥ 4.7, M: ≥ 7.3	5/8			
MA	F: < 9, M: < 36	15/24	> 0.99	NE	–
	F: ≥ 9, M: ≥ 36	21/36			
VAI	F: < 97, M: < 40	28/42	0.1	NE	–
	F: ≥ 97, M: ≥ 40	8/18			
SAI	F: < 127, M: < 28	23/34	0.09	NE	–
	F: ≥ 127, M: ≥ 28	13/26			
VSR	F: < 0.8, M: < 0.6	8/19	0.1	NE	–
	F: ≥ 0.8, M: ≥ 0.6	28/41			

*NE* not entered, *ECOG-PS* Eastern Cooperative Oncology Group performance status, *FEV1* forced expiratory volume in 1 s, *BMI* body mass index, *MA* mean muscle attenuation, *SMI* skeletal mass muscle index, *PMI* psoas muscle index, *VAI* visceral adiposity index, *SAI* subcutaneous adiposity index, *VSR* visceral-to-subcutaneous fat ratio, *CI* confidence interval.

^a^These variables have missing values.

**Figure 2:**
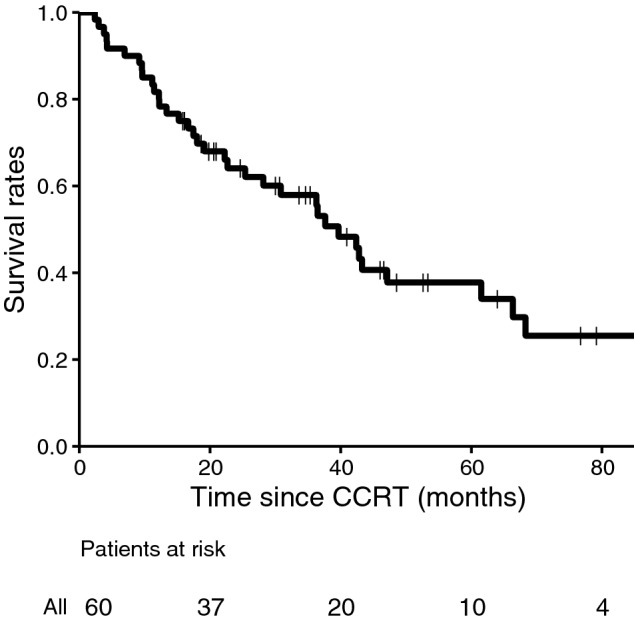
Overall survival curve. Proportion of surviving patients shown according to the number of months after radiotherapy. *CCRT* concurrent chemoradiotherapy.

**Figure 3:**
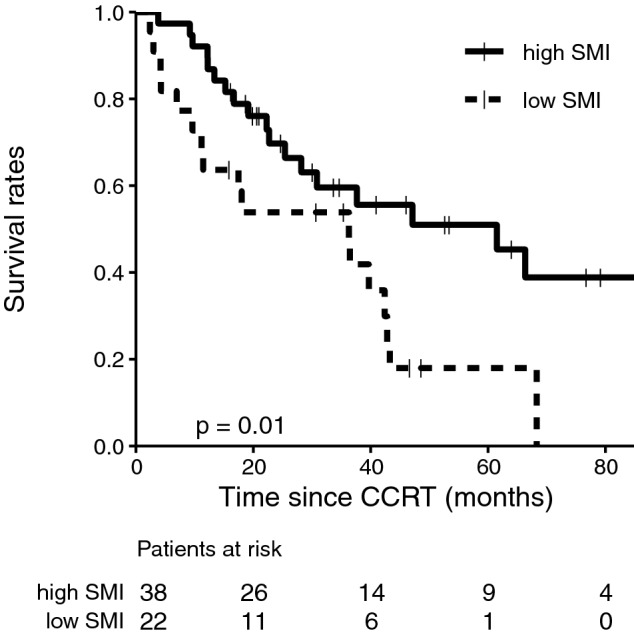
Subgroup analysis of overall survival stratified according to SMI. Proportion of surviving patients shown according to the number of months after radiotherapy, where the filled line represents a low SMI and the dotted line indicates a high SMI. *SMI* skeletal muscle index, *CCRT* concurrent chemoradiotherapy.

Figure 2 shows OS curve for all patients. The 1, 3, and 5-year OS rates were 81.7%, 57.9%, and 37.8%, respectively, for all patients. Further, the 1, 3, and 5-year OS rates were 92.1%, 59.6%, and 51.0% in patients with high SMI, and 63.6%, 53.8%, and 17.9% in patients with low SMI, respectively (Fig. 3). Details of the OS rates for sarcopenia are shown in Table 4. The 1, 3, and 5-year survival rates were 90.5%, 85.4%, and 64.3% for the groups with a PS of 0, and 75.0%, 44.7%, and 25.6% for the groups with a PS of 1, respectively (p = 0.004).

**Table 4:** Overall survival rates for sarcopenia.

year	OS (95% CI) for all patients	OS with sarcopenia (low SMI) (95% CI)	OS without sarcopenia (high SMI) (95% CI)
1	81.7% (72.4–92.1%)	63.6% (46.4–87.3%)	92.1% (83.9–100%)
2	64.1% (52.8–77.7%)	53.8% (36.4–79.7%)	69.7% (56.1–86.6%)
3	57.9% (46.3–72.5%)	53.8% (36.4–79.7%)	59.6% (45.0–78.9%)
4	37.8% (25.9–55.0%)	17.9% (6.6–49.0%)	51.0% (35.7–72.7%)
5	37.8% (25.9–55.0%)	17.9% (6.6–49.0%)	51.0% (35.7–72.7%)

*CI* confidence interval, *OS* overall survival, *SMI* skeletal mass muscle index.

## Discussion

This was the first study to demonstrate that the SMI was associated with OS in patients with stage III NSCLC who underwent definitive CCRT. Studies focusing on the relationship between the SMI and OS in patients with cancer have predominantly been conducted in gastrointestinal cancer settings. Fujiwara et al. reported a significant association of the SMI and MA with mortality in patients with hepatocellular carcinoma, and Okumura et al. showed a relationship of the SMI and MA with OS in patients who underwent surgery for cholangiocarcinoma^25,26^. In terms of lung cancer, several systematic reviews and meta-analyses have focused on the association between sarcopenia and OS after treatment for lung cancer. Buentzel et al. conducted an analysis of 15 studies on patients with lung cancer (including all stages of NSCLC and small-cell lung cancer), and based on the findings of multivariate analysis, reported that patients with lung cancer and sarcopenia had a three-fold greater risk of death than those without sarcopenia^8^. Yang et al. who conducted an analysis of thirteen studies showed that sarcopenia was an independent prognostic factor for poor OS in patients with stage III–IV NSCLC^9^. Those reviews included several treatment modalities, including surgery, stereotactic radiotherapy^10^, chemoradiotherapy, and chemotherapy. Only two studies have considered SMI as an assessment tool for sarcopenia and examined its relationship with OS in patients who were treated with chemoradiotherapy. Kiss et al. reported that 41 patients with stage I–III NSCLC who received CCRT showed a significant decrease in the SMI and MA at 4 weeks after starting treatment and that low MA tended to be associated with poor OS (p = 0.13)^18^. However, the presence of sarcopenia before treatment is not associated with poor OS. Bowden et al. demonstrated that low MA was associated with significantly lower OS rates in patients with lung cancer, including those with NSCLC and small-cell lung cancer, who were treated with chemoradiotherapy^17^. The SMI was not associated with OS in Bowden et al.’s^17^ study as well. In our study, the SMI was a prognostic factor for OS, whereas MA was not. The study by Kiss et al. differs from our study with respect to the following aspects: the HU thresholds for SMI measurements ranged from − 19 to 150, and they included stage I–III cases. The study by Bowden et al. differs from our study with respect to the following aspects: it included sequential chemotherapy cases and CCRT cases, patients with small-cell lung cancer, and patients with all disease stages. In addition, the treatment strategies for stage III disease vary across institutions, with our institution prioritizing preoperative CCRT plus surgery. Therefore, we cannot rule out the possibility that the extent of progression and general condition in our patients may have significantly differed from those in patients in the aforementioned reports.

In our study, we included patients with stage III NSCLC who had CT data pertaining to the L3 level, with no restrictions on the chemotherapy regimen. Thus, our results may have differed from those of previous studies. In our study, the PMI was also not a prognostic factor. Hamaguchi et al. reported that in 541 adult liver donors (living donor liver transplantation), the PMI was positively correlated with the SMI^28^. Theoretically, if the SMI is significantly associated with OS, similar results should have been obtained for the PMI; however, it is not clear why this was not the case. Mitsuyoshi et al. reported that a high PMI was not correlated with high OS (p = 0.873) in 89 patients with stage III NSCLC with sex-specific cutoffs^32^; therefore, the PMI may not be an important parameter in lung cancer. Data on the association of the SMI and PMI with OS in patients with NSCLC are scarce in the setting of CCRT compared with surgery, and thus, further studies are warranted.

Muscle loss is a major symptom of cancer cachexia, along with anorexia, weight loss, anemia, and altered carbohydrate, lipid, and protein metabolism. Cancer cachexia is recognized as a paraneoplastic syndrome in 60–80% of the patients with advanced-stage disease. More than 30% of the patients with cancer die due to cachexia, and more than 50% of them die in the presence of cachexia, which is associated with adverse prognosis and shorter survival times^33,34^. Sarcopenia may be a marker to preemptively predict the development of cancer cachexia. Recently, skeletal muscle has been identified as a secretory organ, which secretes myokines that may affect the growth of cancer cells^35^. Hojman et al. reported that myokines, which are released from muscles during exercise inhibit the growth of breast cancer cells and induce apoptosis of these cells^36^. Therefore, as muscle mass decreases, myokine levels and responses may decrease, making it easier for cancer to progress.

In our study, a higher BMI was correlated with a higher OS rate in the univariate analysis. A high BMI is correlated to poor OS after cancer treatment^19^; however, there is some controversy regarding the same in patients with lung cancer. In their meta-analysis, Gupta et al. showed that patients who were obese in the pre-treatment stage had lower lung cancer mortality rates than those with normal weight; our results showed the same trend. In gastrointestinal cancers, high VAI, SAI, and VSR have been shown to be associated with poor OS rates^25,26^; however, no correlation was found in our study that examined data of patients with lung cancer following CCRT. The reason for this is that despite difficulties in the performance of simple comparisons, the median VAI and SAI values were 31.8 cm^2^/m^2^ and 41.4 cm^2^/m^2^, respectively, in the study by Fujiwara et al., which are higher than the values of 27.0 cm^2^/m^2^ and 28.7 cm^2^/m^2^, respectively, observed in our study. This may be largely attributed to differences in patients’ backgrounds. Regarding OS, the result observed in patients with lung cancer may differ from that in those with other carcinomas with respect to adipose mass, as with BMI. In patients undergoing CCRT for stage III NSCLC, adipose mass reductions are not given high priority, with efforts primarily driven toward increasing or maintaining patients’ muscle mass. To the best of our knowledge, our study was the first to examine the association between post-treatment OS rates for lung cancer and the VAI, SAI, and VSR, as assessed using CT. In our study, the BMI scores (median 21.7) of all patients were normal, unlike that in the study by Martin et al., in which the average BMI scores were 26.0 for men and 25.1 for women^7^. Among patients with lung cancer and normal BMI, adipose mass may not be related to prognosis; although, further studies on adipose mass are warranted.

In the study of Bowden et al., a PS ≥ 2 was associated with poor OS^17^. Similarly, in our study, the PS was a predictor of prognosis, which is a reasonable result.

Exercise is effective in counteracting the catabolic effects of muscle by increasing the protein synthesis rate and decreasing the proteolysis rate owing to its anti-inflammatory effects^37,38^. Exercise training can attenuate or reverse the process of muscle wasting through anti-inflammatory and antioxidant effects that can attenuate the signaling pathways of proteolysis and activate protein synthesis molecules^38^. Gould et al. stated in their review that exercise successfully improved muscle strength and physical function in patients with cachexia in non-cancer-related diseases and that exercise interventions provided at various stages of treatment may improve the protein synthesis rate and help patients recover from low body weight and reverse proteolysis^37^. Delrieu et al. reported that participation in a 6-month personalized walking program resulted in significant improvements in the 6-min walking distance test (+ 7%) and isometric quadriceps strength (+ 22%) among patients with metastatic breast cancer^39^. Exercise interventions may aid in increasing or maintaining the muscle strength in patients with lung cancer. Future clinical trials are warranted to determine whether improvements in muscle strength can lead to OS enhancements.

Our study has some limitations. Since this study was conducted at a single center with a short follow-up period, and CCRT is not the only treatment option for stage III NSCLC, the presence of an unmeasured bias cannot be ruled out. According to the European Working Group on Sarcopenia in Older People guidelines, the diagnosis of sarcopenia requires low muscle strength or low physical performance in addition to low muscle mass; however, because of the retrospective nature of the study, muscle strength measurements could not be performed.

In conclusion, we demonstrated that the SMI is correlated with prognosis in patients with stage III lung cancer undergoing CCRT, whereas adipose mass is not. The additional consideration of sarcopenia improved the predictability degree for OS in our settings, and therefore, could aid in early intervention to maintain muscle mass and improve prognosis.

## Data Availability

The institution’s review board prohibits data sharing.
