# Publisher Correction: Sarcopenia is associated with poor prognosis after chemoradiotherapy in patients with stage III non-small-cell lung cancer: a retrospective analysis

**DOI:** 10.1038/s41598-021-93955-6

**Published:** 2021-07-12

**Authors:** Kuniaki Katsui, Takeshi Ogata, Soichi Sugiyama, Kotaro Yoshio, Masahiro Kuroda, Takao Hiraki, Katsuyuki Kiura, Yoshinobu Maeda, Shinichi Toyooka, Susumu Kanazawa

**Affiliations:** 1 Department of Proton Beam Therapy, Okayama University Graduate School of Medicine, Dentistry and Pharmaceutical Sciences, 2‑5‑1 Shikata‑cho, Kita‑ku, Okayama, 700‑8558 Japan; 2 Department of Radiology, Iwakuni Clinical Center, 1‑1‑1 Atagomachi, Iwakuni, Yamaguchi 740‑8510 Japan; 3 Department of Radiology, Okayama University Hospital, 2‑5‑1 Shikata‑cho, Kita‑ku, Okayama, 700‑8558 Japan; 4 Department of Radiological Technology, Graduate School of Health Sciences, Okayama University, 2‑5‑1 Shikata‑cho, Kita‑ku, Okayama, 700‑8558 Japan; 5 Department of Radiology, Okayama University Graduate School of Medicine, Dentistry and Pharmaceutical Sciences, 2‑5‑1 Shikata‑cho, Kita‑ku, Okayama, 700‑8558 Japan; 6 Department of Allergy and Respiratory Medicine, Okayama University Hospital, 2‑5‑1 Shikata‑cho, Kita‑ku, Okayama, 700‑8558 Japan; 7 Department of Hematology, Oncology and Respiratory Medicine, Okayama University Graduate School of Medicine, Dentistry, and Pharmaceutical Sciences, 2‑5‑1 Shikata‑cho, Kita‑ku, Okayama, 700‑8558 Japan; 8 Department of General Thoracic Surgery and Breast and Endocrinological Surgery, Okayama University Graduate School of Medicine, Dentistry and Pharmaceutical Sciences, 2‑5‑1 Shikata‑cho, Kita‑ku, Okayama, 700‑8558 Japan

Correction to: *Scientific Reports* 10.1038/s41598-021-91449-z, published online 04 June 2021

The original version of this Article contained errors in Table 3, where some values in Number of deaths were incorrectly given. The original Table 3 and accompanying legend appear below.

**Table 3:** Univariate and multivariate analyses of factors associated with overall survival.

Factor		Number of deaths	Univariate analysis	Multivariate analysis	Hazard ratio
			*p*-value	*p*-value	(95% CI)
Sex	F	04-Sep	0.3	NE	–
	M	32/51			
Age (years)	< 66	16/28	0.6	NE	–
	≥ 66	20/32			
T Stage	X, 1–2	Oct-22	0.2	NE	–
	3–4	26/38			
N Stage	0–1	03-Aug	0.4	NE	–
	2–3	33/52			
Clinical stage	IIIA	Jun-16	0.3	NE	–
	IIIB	22/32			
	IIIC	08-Dec			
Histology	Adenocarcinoma	16/26	0.5	NE	–
	Others	20/34			
Smoking history^a^	Never/former	21/36	0.9	NE	–
	Current	13/22			
ECOG-PS^a^	0	Aug-21	0.004	0.011	2.91
	1	25/36			(1.28–6.64)
Laterality	Right	17/31	0.5	NE	–
	Left	18/26			
Lobe	Upper/middle	28/49	0.2	NE	–
	Lower	08-Nov			
FEV1 (l)^a^	< 2.2	17/29	0.2	NE	–
	≥ 2.2	13/22			
Chemotherapy	Standard	29/49	0.5	NE	–
	Reduced	07-Nov			
BMI^a^	F: < 17, M: < 25	28/41	0.03	NE	–
	F: ≥ 17, M: ≥ 25	Jul-18			
SMI	F: < 24, M: < 43	17/22	0.01	0.02	2.36
	F: ≥ 24, M: ≥ 43	19/38			(1.15–4.85)
PMI	F: < 4.7, M: < 7.3	31/52	0.4	NE	–
	F: ≥ 4.7, M: ≥ 7.3	05-Aug			
MA	F: < 9, M: < 36	15/24	> 0.99	NE	–
	F: ≥ 9, M: ≥ 36	21/36			
VAI	F: < 97, M: < 40	28/42	0.1	NE	–
	F: ≥ 97, M: ≥ 40	Aug-18			
SAI	F: < 127, M: < 28	23/34	0.09	NE	–
	F: ≥ 127, M: ≥ 28	13/26			
VSR	F: < 0.8, M: < 0.6	Aug-19	0.1	NE	–
	F: ≥ 0.8, M: ≥ 0.6	28/41			

*NE* not entered, *ECOG-PS* Eastern Cooperative Oncology Group performance status, *FEV1* forced expiratory volume in 1 s, *BMI* body mass index, *MA* mean muscle attenuation, *SMI* skeletal mass muscle index, *PMI* psoas muscle index, *VAI* visceral adiposity index, *SAI* subcutaneous adiposity index, *VSR* visceral-to-subcutaneous fat ratio, *CI* confidence interval.

^a^These variables have missing values.

The original Article has been corrected.

